# EVALUATING THE FEASIBILITY AND EFFICACY OF A PILOT INTERVENTION TARGETING UNEMPLOYED OLDER WORKERS

**DOI:** 10.1093/geroni/igz038.476

**Published:** 2019-11-08

**Authors:** Jie Yang

**Affiliations:** East Carolina University School of Social Work, Greenville, North Carolina, United States

## Abstract

The great recession of 2007–2009 has led to historically high unemployment rates, and almost half of the unemployed population was long-term unemployed in 2010. Both discriminations against the long-term unemployed and ageism hinders older unemployed workers reentering the workforce, and they risk severe mental health deterioration. However, traditional job training programs are shown to be ineffective in protecting their participants’ mental health. Hence, adopting Jahoda’s Latent Deprivation Theory, the author worked with a non-profit in the Greater Boston area on developing an innovative intervention targeting older unemployed older workers’ mental health and income generation outcomes. The pilot intervention (lasting three months) created a quasi-office environment where participants can have a time structure, engage with peers, work on projects, and receive training. The current study evaluates the effect of this pilot program on multiple mental health outcomes. Pre-and-post comparisons were conducted using quantitative analysis (N=13). Participants’ depressive symptoms dropped significantly, and they reported having a better time structure of the day as well as higher scores in extraversion in terms of personality change. Qualitative analyses were conducted to analyze the pre-and-post interviews. Participants reported overwhelming approval of the intervention in helping them better cope with unemployment and being productive in seeking jobs or generating incomes. One example of quotes is “…compared to three months ago, I’m in a very different place. Before the Collaboratory, I had become extremely depressed and withdrawn…The Collaboratory has helped make a massive difference in my sense of well being right now.”

